# Multilevel analysis of anemia and associated factors among women of reproductive age (15–49 years) in Liberia: Evidence from the 2019/20 Liberia demographic and health survey data

**DOI:** 10.1371/journal.pone.0296747

**Published:** 2024-04-25

**Authors:** Dagnew Getnet Adugna, Misganaw Asmamaw Mengstie, Fitalew Tadele Admasu, Maritu Gebnie Teshome, Hailu Aragie, Tadesse Asmamaw Dejenie

**Affiliations:** 1 Department of Human Anatomy, School of Medicine, College of Medicine and Health Science, University of Gondar, Gondar, Ethiopia; 2 Department of Biochemistry, College of Medicine and Health Science, Debre Tabor University, Debre Tabor, Ethiopia; 3 Department of Clinical Midwifery, School of Midwifery, College of Medicine and Health Science, University of Gondar, Gondar, Ethiopia; 4 Department of Biochemistry, School of Medicine, College of Medicine and Health Science, University of Gondar, Gondar, Ethiopia; Universite de Kinshasa, THE DEMOCRATIC REPUBLIC OF THE CONGO

## Abstract

**Background:**

Anemia is a global public health problem, principally affecting young children and reproductive-age mothers. Although anemia is a main public health concern in low-income countries, there is no evidence about its prevalence and associated factors among women of reproductive age in Liberia. Thus, the purpose of this study was to identify the prevalence and associated factors of anemia among women of reproductive age in Liberia.

**Methods:**

We used the data extracted from the fifth Liberia Demographic and Health Survey (LDHS-V) that were carried out between October 2019 and February 2020. The sample was chosen using a stratified two-stage cluster sampling procedure. Overall weighted samples of 4027 women of reproductive age were used in the analysis. Data weighting was carried out to obtain reliable estimates and standard errors as well as to restore the representativeness of the data. Stata version 14 software was used for data extraction, coding, and analysis. We used multilevel analysis to identify the significant factors associated with anemia among women of reproductive age.

**Results:**

The prevalence of anemia among women of reproductive age in Liberia was 44.51 (95% CI: 42.97–46.04). From these, about 23.10% of women of reproductive age were mildly anemic, 20.63% were moderately anemic and 0.78% was severely anemic. In multivariable analysis; women with the groups of 20–24 years (adjusted odds ratio (AOR) = 0.72, 95% CI: 0.56, 0.92), 25–29 years (AOR = 0.57, 95% CI: 0.43, 0.77), 30–34 years (AOR = 0.59, 95% CI: 0.43, 0.83), 35–39 years (AOR = 0.56, 95% CI: 0.41, 0.79), 40–44 years (AOR = 0.61, 95% CI: 0.43,0.87), 45–49 years (AOR = 0.57, 95% CI: 0.39,0.82), overweight (AOR = 0.83; 95% CI: 0.70, 0.98), obese (AOR = 0.72; 95% CI: 0.58, 0.88), using modern contraceptive methods (AOR = 0.61; 95% CI: 0.52, 0.72), and being from the Northcentral region (AOR = 0.55; 95% CI: 0.43, 0.72) were significantly associated with lower odds of anemia. However, being pregnant (AOR = 1.34; 95% CI: 1.04, 1.73) and having higher parity (3 children or more) (AOR = 1.40; 95% CI: 1.03, 1.93) were significantly associated with higher odds of anemia.

**Conclusion:**

In the present study, the prevalence of anemia in women of reproductive age was relatively high. Therefore, it is better to provide special emphasis on high-risk groups such as pregnant and multiparous women.

## Introduction

Anemia is a disorder in which the number of erythrocyte cells (hemoglobin levels) is inadequate to meet the physiologic desires of the body tissue [1]. It is a main worldwide public health problem, principally affecting young children and reproductive-age mothers [2–4]. Globally, over 500 million (33%) women of reproductive age suffer from anemia which has a long-term negative impact on both the health of mothers and their children as well as economic development [5]. However, the highest-burden (49.7%) of anemia in women of reproductive age is found in sub-Saharan Africa [6].

Anemia has significant long-term adverse impacts on the health of general populations; especially women are among the vulnerable groups because of their experiences of menstruation, pregnancy, and childbirth-related hemorrhage [7]. Anemia in women of childbearing age causes low productivity because of decreased work capacity, high infection risk because of its effect on immunity, termination of pregnancy, and maternal death [8–11]. Furthermore, maternal anemia is associated with adverse neonatal health outcomes like premature birth, mental retardation, small birth weight, and decreased baby iron stores, which may ultimately lead to child death [10–13].

Even though the etiologies of anemia are multifactorial, it may be caused by nutritional and non-nutritional causes [14–16]. Due to the high demand for iron during pregnancy, breastfeeding, and menstrual period, iron deficiency is the most common cause of anemia in women of childbearing age [10, 17].

Previous studies found that different individual and community-level factors are significantly associated with anemia among women of reproductive age. The individual-level factors include maternal-related factors (age [5, 18, 19], level of education [5, 20, 21], occupational status [5, 22, 23], marital status [5, 24–26], women’s body mass index [10, 20, 24, 26], ever having terminated pregnancy [12, 27, 28], parity [5, 29], family size [5, 30–32], modern contraceptive use [5, 24, 32], current pregnancy status [19–21, 26, 33], currently breastfeeding [21, 27]), and household related factors (wealth index [5, 20, 21, 24, 29, 33], sex of household head [5, 19, 22, 34, 35], exposure to media [5, 27, 36], type of toilet facility [5, 6, 20, 33], source of drinking water [5, 19, 20, 33]). Moreover, community-level factors were place of residence [5, 37], and community literacy level [5, 22].

The world health organization (WHO) considers anemia a serious public health problem when its prevalence is above 5% [38]; however, the majority of the evidence shown above indicates that the burden of anemia among mothers of reproductive age is greater than 20%. WHO has established a worldwide aim of accomplishing a 50% decrease in anemia prevalence among women of reproductive age by 2025 [11], even though it is difficult to achieve this aim in the recent trend. Although anemia is a main public health concern in low-income countries, there is no evidence about its prevalence and associated factors among women of reproductive age in Liberia. Thus, the purpose of this study was to identify the prevalence and associated factors of anemia among women of reproductive age in Liberia.

## Methods and materials

### Data source, sampling technique, and population

We used the data extracted from the fifth Liberia Demographic and Health Survey (LDHS-V) that was carried out between October 2019 and February 2020. The LDHS has performed a stratified, two-stage cluster sampling technique. In the first stage, a total of 325 clusters were selected using a stratified two-stage cluster sampling technique. In the second stage, a fixed number of households (30 households for each cluster) were selected using a systematic sampling technique. For this study, we used the woman’s data (IR) file and an overall weighted sample of 4027 women of reproductive age.

### Variables of the study

#### Outcome variable

The dependent variable for this study was anemia level, which was determined by the mother’s pregnancy status; when non-pregnant a hemoglobin level <12.0 g per deciliter (g/dl), and when pregnant a hemoglobin concentration <11.0 g/dl were considered as anemia. Based on severity, anemia was also categorized as mild (if hemoglobin levels were between 10.0 and 10.9 g/dl and 10.0 and11.9 g/dl for pregnant women and non-pregnant women, respectively); moderate (if hemoglobin values were between 7.0 and 9.9 g/dl); and severe (if hemoglobin level <7.0 g/d) for both pregnant and non-pregnant women. In our study, we re-classified anemia status as anemic, which was coded as “1” and non-anemic, coded as “0”.

#### Independent variables

According to different literature, the explanatory variables included in the study were individual-level and community-level factors. Individual-level variables considered were classified as maternal-related factors and household-related factors. The maternal-related factors were age of the mothers (categorized as 15–19 years, 20–24 years, 25–29 years, 30–34 years, 35–39 years, 40–44 years, and 45–49 years), educational status (no primary education, primary education, and secondary and above), occupational status (working and not working), marital status, having ever had a terminated pregnancy (yes and no), parity, perception of distance from the health facility, modern contraceptive use, current pregnancy (yes and no) status, breastfeeding, body mass index, and respondents slept under the mosquito net. The household-level factors include wealth index (poorest, poorer, middle, richer, and richest), sex of household head, household size, media exposure (made from 3 factors: frequency of listening to radio, frequency of watching television, and frequency of reading newspapers), type of toilet facility (improved and non-improved), and source of drinking water. The community level factors were residence (urban and rural) and region (Northwestern, Southcentral, Southeastern-a, Southeastern-b, and Northeastern).

### Data management and analysis

Data extraction, coding, and analysis were done using Stata version 14 software. Data weighting was carried out throughout the analysis to obtain reliable estimates and standard errors as well as to restore the representativeness of the data. Descriptive statistics were done using frequencies and percentages. A multilevel binary logistic regression model was fitted to determine associated factors of anemia because of the hierarchical nature of LDHS data. In LDHS, study participants were nested within clusters, and we assume that participants within the same cluster are more likely to share similar characteristics than participants in another cluster. The independent and equal variance assumptions of the traditional logistic regression model are violated in this situation. As a result, a sophisticated model must be used to account for the heterogeneity between clusters. Four models were developed during the multilevel analysis: the first (null model), which only incorporated the dependent variable; the second (Model I), which only included individual-level factors; the third (Model II), which only included community-level variables; and the fourth (Model III), which included both individual and community-level variables. To detect the clustering effect or variability, the intraclass correlation coefficient (ICC), median odds ratio (MOR), and proportional change in variance (PCV) were checked. Model comparison was done using deviance (-2 log-likelihood (LL)), and the model with the lowest deviance was declared to be the best-fitted model. To select the variables for the multivariable logistic regression analysis, a binary bivariable logistic regression analysis was initially performed, and variables with a p-value of less than 0.20 were selected as candidates for the multivariable logistic regression analysis. Variables with a P-value of less than 0.05 in the multivariable logistic regression analysis were considered significant factors associated with anemia among women of reproductive age, and an adjusted odds ratio (AOR) with a 95% confidence interval (CI) was reported.

### Ethical considerations

Ethical approval and participant consent were not required for this study because it was a secondary data analysis of publically accessible survey data from the MEASURE DHS Program. The authors requested DHS Program and permission was obtained to download and utilize the data for this study from https://www.dhsprogram.com/data/dataset_admin/login_main.cfm. The datasets contain neither household addresses nor names of individuals. There are no names of participants or household addresses recorded in these data sets.

## Results

### Sociodemographic characteristics of women in Liberia

An overall weighted sample of 4027 reproductive-age women (15–49 years) was included in the final analysis. The mean [±SD] age of the women was 29.2 (±10.01) years. The highest proportion (21.66%) of women was in the age group of 15–19 years and nearly 39.26% of the respondents were unmarried. About 45.87% of respondents had secondary education and above. Around 64.77% of women had media exposure, and 63.04% of them had no job or were currently not working. Most (84.74%) of women were from households with an improved source of drinking water, and about half (50.98%) were from households with an unimproved type of toilet facility. About three-quarters (74.60%) of women did not use modern contraceptives. Concerning residence and sex of household heads, about 62.46% and 60.02% of respondents were urban dwellers and male-headed households, respectively. Regarding region, about half (50.70%) of respondents were from the Southcentral region (Table 1).

**Table 1:** Socio-demographic characteristics of reproductive-age women in Liberia (N = 4027).

Variables	Categories	Frequency	Percentage
**Individual level variables**			
Age (years)	15–19	872	21.66
	20–24	755	18.74
	25–29	637	15.82
	30–34	514	12.76
	35–39	523	12.98
	40–44	386	9.58
	45–49	340	8.45
Occupation	Working	1488	36.96
	Not working	2539	63.04
Marital status	Never in union	1581	39.26
	Married	990	24.58
	Divorced/separated/widowed	1456	36.16
Educational status	No education	1211	30.08
	Primary education	969	24.05
	Secondary and above	1847	45.87
Sex of household head	Male	2417	60.02
	Female	1610	39.98
Household wealth status	Poorest	708	17.58
	Poorer	713	17.72
	Middle	812	20.16
	Richer	899	22.32
	Richest	895	22.22
Media exposure	No	1419	35.23
	Yes	2608	64.77
Type of toilet facility	Improved	1974	49.02
	Non-improved	2053	50.98
Source of drinking water	Improved	3413	84.74
	Non-improved	614	15.26
Household size	1–2	338	8.39
	3–5	1472	36.56
	≥ 6	2217	55.05
Ever had a terminated pregnancy	No	3258	80.90
	yes	769	19.10
Distance from the health facility	Big problem	1144	28.41
	Not a big problem	2883	71.59
Body mass index (Kg/m2)	Underweight	205	5.10
	Normal	2164	53.73
	Overweight	996	24.73
	Obesity	662	16.44
Modern contraceptive use	Yes	1023	25.40
	No	3004	74.60
Currently pregnant	No or unsure	3746	93.01
	Yes	281	6.99
Currently breastfeeding	No	3147	78.15
	Yes	880	21.85
Parity	None	934	23.20
	1–2	1365	33.90
	≥3	1728	42.90
Respondent slept under a mosquito bed net	No	2321	57.63
	Yes	1706	42.37
**Community level variables**			
Residence	Urban	2515	62.46
	Rural	1512	37.54
Region	Northwestern	297	7.38
	Southcentral	2042	50.70
	Southeastern a	235	5.85
	Southeastern b	222	5.51
	Northcentral	1231	30.56

### Anemia prevalence among women of childbearing age (15–49 years) in Liberia

**Fig 1:**
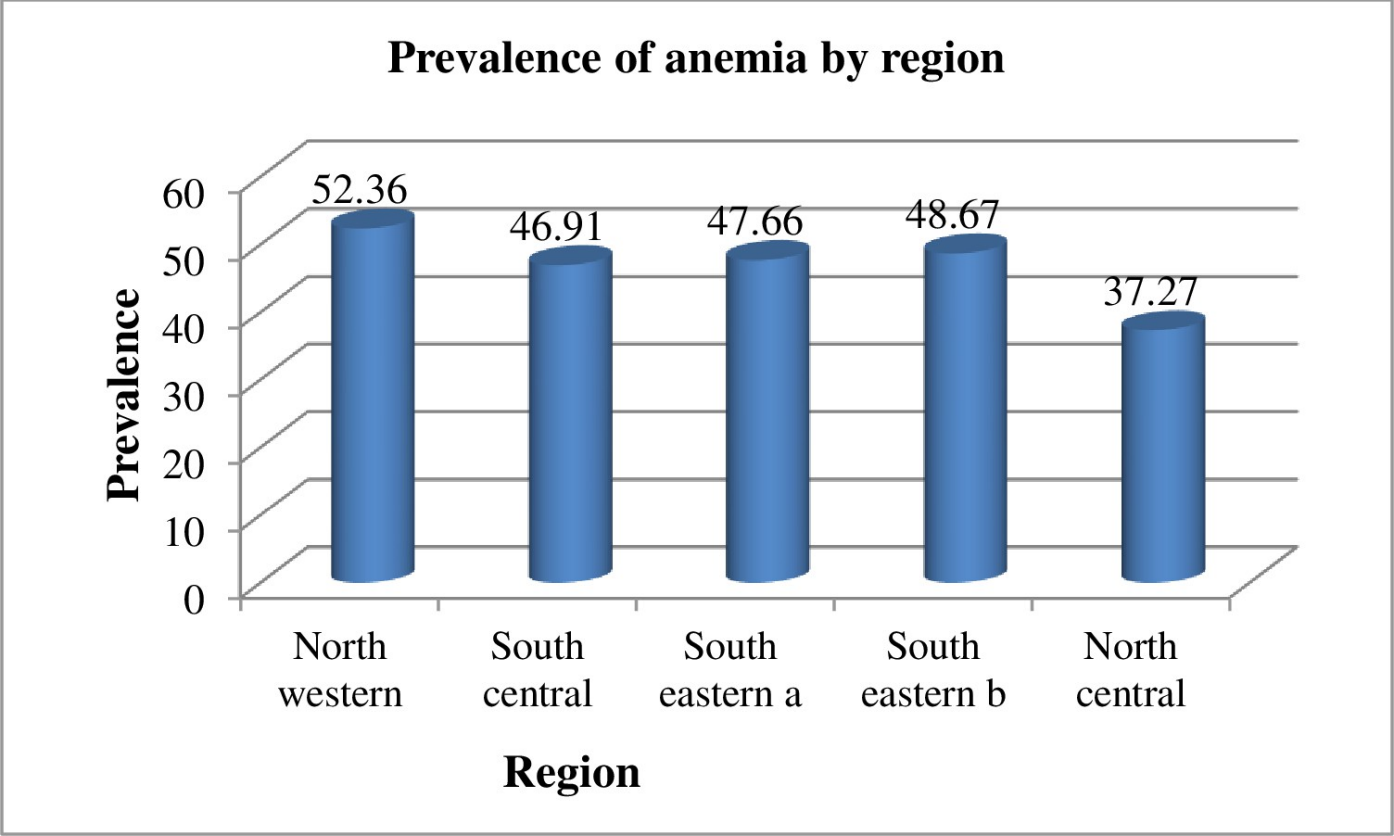
The prevalence of anemia among women of reproductive age in Liberia.

In our study, the prevalence of anemia among women of reproductive age in Liberia was 44.51% (95% CI: 42.97–46.04). The study revealed that 23.10% of women of reproductive age had mild anemia, 20.63% had moderate anemia, and 0.78% had severe anemia. The prevalence of anemia was higher in women from the Northwestern region (52.36%) and lower in those from the Northcentral region (37.27%) (Fig 1).

### Results of the random effect analysis and model selection

In this study, ICC, MOR, and PCV were used to assess the random-effects model analysis. The community-level variability was measured by both ICC and MOR. The ICC value in the null model was 5.9%, revealing that 5.9% of the total variability of the level of anemia in women of reproductive age was because of differences between clusters whereas the remaining unexplained 94.1% of the total variability of the level of anemia was due to individual differences. Additionally, the highest MOR value (1.16) in the null model supports the fact that there was significant clustering of anemia in women of reproductive age. Moreover, the highest PCV value (0.28) in the last model (model III) indicated that 28% of the variation in anemia among reproductive-age women was explained by both individual-level and community-level variables. The final model (model III), which contains both individual and community-level factors simultaneously, was chosen as the best-fitted model for the data as it had the lowest deviance value (5432.10). We used the last model to identify the significant factors associated with anemia among women of reproductive age in Liberia (Table 2).

**Table 2:** The results of random effect analysis and model fitness for the assessment of anemia among women of reproductive (15–49 years) in Liberia.

Parameter		Null model	Model I	Model II	Model III
Community level variance		0.21(0.13–0.31)	0.20(0.12–0.33)	0.16(0.10–0.26)	0.15(0.09–0.26)
ICC		0.059	0.057	0.046	0.044
MOR		1.16	1.14	1.03	1.01
PCV		Ref	0.047	0.23	0.28
Model comparison					
LLR	-2791.39		-2732.86	-2778.43	-2716.0511
Deviance (-2LLR)	5582.78		5465.72	5556.86	5432.10

ICC = Intra-class Correlation Coefficient: LLR = Log-likelihood Ratio: MOR: Median Odds Ratio

### Factors associated with anemia among women aged 15–49 years in Liberia

In the bivariate analysis, all variables (except occupational status, marital status, sex of household head, source of drinking water, having ever had a terminated pregnancy, and distance from a health facility) had p-value <0.2 and were considered for multivariable analysis. In the multilevel multivariable regression analysis; both individual-level variables (age, body mass index, modern contraceptive use, parity, and current pregnancy status) and community-level variables (region) were found to be significant factors of anemia in reproductive-age women (Table 3). The odds of developing anemia in the older age groups of 20–24 years, 25–29 years, 30–34 years, 35–39 years, 40–44 years, and 45–49 years were decreased by 28% (adjusted odds ratio (AOR) = 0.72, 95% CI: 0.56, 0.92), 43% (AOR = 0.57, 95% CI: 0.43, 0.77), 41% (AOR = 0.59, 95% CI: 0.43, 0.83), 44% (AOR = 0.56, 95% CI: 0.41, 0.79), 39% (AOR = 0.61, 95% CI: 0.43,0.87), and 43% (AOR = 0.57, 95% CI: 0.39,0.82) compared to the age group 15–19 years, respectively. Overweight and obese women had lower odds of developing anemia by 17% (AOR = 0.83; 95% CI: 0.70, 0.98) and 28% (AOR = 0.72; 95% CI: 0.58, 0.88), respectively, compared with women with normal body weight. A woman who used modern contraceptive methods had reduced odds of developing anemia by 39% (AOR = 0.61; 95% CI: 0.52, 0.72) compared to women who did not use any contraceptives. The odds of having anemia among currently pregnant women were 1.34 times (AOR = 1.34; 95% CI: 1.04, 1.73) higher as compared to non-pregnant women. Regarding parity, odds of having anemia in women with higher parity (more than 2 children) were 1.40 (AOR = 1.40; 95% CI: 1.03, 1.93) times higher than women with no children. Furthermore, being from the Northcentral region was associated with a 45% lower prevalence of anemia as compared to the Southeastern b region (AOR = 0.55; 95% CI: 0.43, 0.72) (Table 3).

**Table 3:** Shows bivariable and multivariable multilevel regression analysis to determine factors associated with anemia among reproductive-age women in Liberia (N = 4027).

Variables	Categories	Anemia		COR (95%CI)	AOR (95%CI)	
		No	Yes			
Age (years)	15–19	392	480	1	1	
	20–24	432	323	0.70 (0.58–0.87)*	0.72(0.56–0.92)*	
	25–29	406	232	0.62 (0.50–0.78)*	0.57(0.43–0.77)**	
	30–34	309	205	0.69(0.55–0.86)*	0.59(0.43–0.83)*	
	35–39	288	235	0.67(0.54–0.84)**	0.56(0.41–0.79)*	
	40–44	214	171	0.72(0.57–0.92)	0.61(0.43–0.87)*	
	45–49	194	146	0.69(0.54–0.89)*	0.57(0.39–0.82)*	
Educational status	No education	690	521	1	1	
	Primary education	496	473	1.09 (0.93–1.28)	1.01(0.84–1.20)	
	Secondary and above	1049	798	0.84(0.71–0.98)*	0.86(0.71–1.05)	
Household wealth status	Poorest	348	360	1	1	
	Poorer	417	296	0.79(0.66–0.95)*	0.86(0.71–1.05)	
	Middle	456	356	0.92(0.75–1.14)	1.03(0.83–1.28)	
	Richer	515	384	0.83(0.66–1.04)	0.96(0.72–1.29)	
	Richest	498	397	0.71(0.55–0.91)*	0.87(0.62–1.22)	
Media exposure	No	795	623	1.15(1.01–1.31)*	1.07(0.93–1.23)	
	Yes	1440	1169	1	1	
Type of toilet facility	Improved	1135	839	1	1	
	Non-improved	1100	953	1.21(1.05–1.40)*	1.14(0.97–1.35)	
Household size	1–2	193	144	1	1	
	3–5	809	664	1.18(0.92–1.52)	1.15(0.89–1.49)	
	≥ 6	1233	984	1.16(0.91–1.49)	1.13(0.87–1.46)	
Body mass index	Underweight	96	110	1.09(0.82–1.46)	1.01(0.74–1.35)	
	Normal	1149	1014	1	1	
	Overweight	593	403	0.78(0.67–0.91)*	0.83(0.70–0.98)*	
	Obesity	397	265	0.68(0.57–0.83)**	0.72(0.58–0.88)*	
Modern contraceptive use	Yes	670	353	0.59(0.52–0.69)**	0.61(0.52–0.72)**	
	No	1565	1439	1	1	
Currently pregnant	No or unsure	2099	1647	1	1	
	Yes	136	145	1.50(1.18–1.89)*	1.34(1.04–1.73)*	
Currently breastfeeding	No	1774	1373	1	1	
	Yes	461	419	1.18(1.01–1.36)*	1.10 (0.92–1.31)	
Parity	No children	458	476	1	1	
	1–2	797	568	0.83(0.69–0.99)	1.15(0.90–1.47)	
	>2	980	748	0.85(0.72–1.01)	1.40(1.03–1.92)*	
Respondent slept under a mosquito bed net	No	1258	1063	1.11(0.97–1.26)	1.09(0.95–1.25)	
	Yes	977	729	1	1	
**Community level variables**						
Residence	Urban	1428	1087	1		1
	Rural	807	705	1.11 (0.94–1.31)		0.94(0.77–1.15)
Region	Northwestern	141	156	1.14 (0.77–1.69)		1.03(0.78–1.36)
	Southcentral	1084	958	0.95(0.69–1.31)		1.00(0.78–1.28)
	Southeastern a	123	112	0.95(0.64–1.43)		0.89(0.68–1.17)
	Southeastern b	114	108	1		1
	Northcentral	772	459	0.63(0.45–0.87)*		0.55(0.43–0.72)**

* P-value < 0.05

** p-value <0.001: COR = Crude odds ratio: AOR = Adjusted Odds Ratio: CI = Confidence Interval: 1 = reference

## Discussion

Anemia among women of reproductive age is a significant public health concern in developing countries due to their increased need for iron during pregnancy, breastfeeding, and menstrual blood loss [10]. In the current study, the prevalence of anemia among women of reproductive age in Liberia was 44.51% (95% CI: 42.97–46.04), which is consistent with a systematic review conducted in developing countries [39]. The prevalence of anemia in this study was higher than in a previous study conducted in Ethiopia [40], Rwanda [24], Democratic Republic of Congo [19], East Africa [5], Nepal [25], and South and Southeast Asian countries [41]. However, the prevalence in this study was lower than in studies carried out in India [42] and Vietnam [43]. The variation in anemia prevalence across countries is likely due to the differences in sociocultural, geographical, and dietary-related factors between countries. Moreover, the high burden of anemia among mothers in Liberia might be due to their social and biological vulnerability to anemia. Furthermore, in low-income countries, particularly Liberia, access to iron-rich food is insufficient because of their low socioeconomic status and limited access to and underutilization of health care, which may contribute to anemia.

Our study indicated that respondent age, body mass index, modern contraceptive use, current pregnant status, parity, and being from the Northcentral region of Liberia were significantly associated with anemia. Older age groups of women had lower odds of anemia than younger age groups (15–19 years). This finding is in agreement with different studies done elsewhere [5, 18, 19, 44]. An increased risk of anemia in relatively younger women might be because of the adverse effects of poor dietary iron intake and the increased demand for iron imposed by iron loss during menstrual blood loss, pregnancy, and lactation [17]. We also found that overweight and obese women had lower odds of anemia as compared to women with normal body weight, and this is in agreement with other studies [24, 25, 41, 45]. A study conducted in China indicated that overweight and obese mothers had higher iron consumption rates than normal body weight mothers [46]. Previous studies revealed that a higher socioeconomic status is associated with good nutritional status [47], preventing infection [48], better access to health care services, and improving other living conditions [47, 49], all of which in turn increase iron intake and prevent anemia.

This study also showed that the use of modern contraceptive methods was significantly associated with anemia. A woman who used modern contraceptive methods had a lower risk of developing anemia as compared to women who did not use any contraceptive, and this is supported by different studies [5, 6, 24, 25]. This might be due to the preventive effects of modern contraceptives on menstrual blood loss, pregnancy, and birth-related complications, which in turn, reduce the burden of anemia due to recurrent blood loss [50, 51]. Simultaneous iron supplementation is also obtained, especially in those mothers who have taken oral hormonal contraceptives, and this could prevent anemia [52].

In this study, we also found that pregnant women had higher odds of anemia as compared with non-pregnant women, and this is in agreement with other studies reported elsewhere [5, 19, 21, 26, 33]. This is because pregnant women need more iron to support their intrauterine fetal development. The second probable reason could be that anemia during pregnancy may result from micronutrient deficiencies, infections, or genetic disorders of the erythrocytes such as thalassemia; all of these are common during pregnancy [53]. Women with a higher parity (3 children or more) had a higher odds of developing anemia as compared to women with no children and this is consistent with other previous studies done elsewhere [54, 55]. This is because the prevalence of anemia increases with the number of pregnancies.

Furthermore, in our study, the region was significantly associated with anemia among women of reproductive age. The odds of anemia were lower among women who were living in the Northcentral region as compared to women from the Southeastern-b region of Liberia. The first possible explanation for the difference in the proportion of anemia could be variation in sociocultural status, availability and accessibility of health care services, economic status, and dietary-related factors between regions within the same country [56]. Additionally, variation in the prevalence of anemia could be due to the discrepancy in the proportion of women taking iron supplements and getting dewormed between regions [57].

## Strengths and limitations of the study

The study has several strengths. Firstly, it was based on a large weighted sample size of nationally representative data. Secondly, appropriate statistical analysis was performed using multilevel analysis to consider the hierarchical nature of the LDHS data and get a reliable estimate. Thirdly, we strongly believe that the study has the potential to provide insight for policymakers and program managers to design appropriate intervention strategies for the problem both at regional and national levels since it is based on the national survey data. Conversely, the study has limitations. Since LDHS data was based on participants’ self-report of variables, there might be a probability of recall bias. Also, since this study was cross-sectional, it is difficult to show the temporal relationship between outcome and explanatory variables. We didn’t address some independent variables like parasitic infection (such as malaria and intestinal parasitic infestation) in the analysis since these variables are not available in the LDHS data.

## Conclusion

In the present study, the prevalence of anemia in women of reproductive age was relatively high. We found that older age, a higher body mass index, the use of modern contraceptive methods, and being from the Northcentral region of Liberia were significantly associated with lower odds of anemia in women of reproductive age. However, being pregnant and having higher parity were significantly associated with a higher prevalence of anemia. Therefore, it is better to provide special emphasis on high-risk groups such as pregnant and multiparous women.

## Supporting information

S1 Checklist *PLOS ONE* clinical studies checklist. (DOCX)

## Abbreviations

AOR: Adjusted odds ratio
CI: Confidence Interval
COR: Crude odds ratio
LDHS: Liberia Demographic Health Survey
ICC: Intraclass Correlation Coefficient
LL: Likelihood
MOR: Median Odds Ratio
PCV: Proportional Change in Variance
WHO: World Health Organization
